# Dickkopf-3 is upregulated in osteoarthritis and has a chondroprotective role

**DOI:** 10.1016/j.joca.2015.11.021

**Published:** 2016-05

**Authors:** S.J.B. Snelling, R.K. Davidson, T.E. Swingler, L.T.T. Le, M.J. Barter, K.L. Culley, A. Price, A.J. Carr, I.M. Clark

**Affiliations:** † Nuffield Department of Orthopaedics, Rheumatology and Musculoskeletal Sciences, University of Oxford, Oxford, UK; ‡ School of Biological Sciences, University of East Anglia, Norwich, UK; § Institute of Cellular Medicine, Newcastle University, Newcastle, UK; ∥ Hospital for Special Surgery and Weill Cornell Medical College, New York, NY, USA

**Keywords:** Cartilage, Wnt, Dickkopf, TGFβ, Osteoarthritis

## Abstract

**Objective:**

Dickkopf-3 (Dkk3) is a non-canonical member of the Dkk family of Wnt antagonists and its upregulation has been reported in microarray analysis of cartilage from mouse models of osteoarthritis (OA). In this study we assessed Dkk3 expression in human OA cartilage to ascertain its potential role in chondrocyte signaling and cartilage maintenance.

**Methods:**

Dkk3 expression was analysed in human adult OA cartilage and synovial tissues and during chondrogenesis of ATDC5 and human mesenchymal stem cells. The role of Dkk3 in cartilage maintenance was analysed by incubation of bovine and human cartilage explants with interleukin-1β (IL1β) and oncostatin-M (OSM). Dkk3 gene expression was measured in cartilage following murine hip avulsion. Whether Dkk3 influenced Wnt, TGFβ and activin cell signaling was assessed in primary human chondrocytes and SW1353 chondrosarcoma cells using qRT-PCR and luminescence assays.

**Results:**

Increased gene and protein levels of Dkk3 were detected in human OA cartilage, synovial tissue and synovial fluid. *DKK3* gene expression was decreased during chondrogenesis of both ATDC5 cells and humans MSCs. Dkk3 inhibited IL1β and OSM-mediated proteoglycan loss from human and bovine cartilage explants and collagen loss from bovine cartilage explants. Cartilage *DKK3* expression was decreased following hip avulsion injury. TGFβ signaling was enhanced by Dkk3 whilst Wnt3a and activin signaling were inhibited.

**Conclusions:**

We provide evidence that Dkk3 is upregulated in OA and may have a protective effect on cartilage integrity by preventing proteoglycan loss and helping to restore OA-relevant signaling pathway activity. Targeting Dkk3 may be a novel approach in the treatment of OA.

## Introduction

Osteoarthritis (OA) is characterized by loss of articular cartilage, joint pain and instability. The mechanisms regulating disease pathogenesis remain elusive with a combination of genetic, inflammatory, mechanical and metabolic factors implicated^1^, ^2^, ^3^.

Chondrocytes from OA cartilage exhibit a disrupted phenotype, hallmarks of which include; altered synthesis of extracellular matrix (ECM) and ECM-degrading enzymes, altered cell signaling activity and increased proliferation^4^. Dysregulation of cell signaling pathways likely contributes to OA pathogenesis by reducing the chondrocyte's ability to maintain cartilage integrity, leading to or exacerbating the phenotypic shift associated with OA. The Wnt and transforming growth factor beta (TGFβ) signaling pathways have been strongly implicated in OA pathogenesis^5^, ^6^.

Dickkopf-3 (Dkk3) is a structurally and functionally divergent member of the Dkk family of Wnt antagonists. Dkk3 activates or inhibits Wnt signaling in a tissue-dependent manner and its impact on cartilage Wnt signaling is unknown^7^, ^8^, ^9^. Dkk3 is a tumour suppressor that inhibits proliferation of cancer cells and is downregulated in several types of human cancer^8^, ^9^, ^10^. It can modulate inflammatory cell activity, maintain tissue organisation via TGFβ signaling and can protect against myocardial infarction-induced fibrosis^11^, ^12^, ^13^, ^14^.

The function of Dkk3 in other tissues suggests it could be an important mediator of chondrocyte homeostasis and maintenance of cartilage integrity. Several studies using animal models of OA have reported increased Dkk3 in diseased cartilage^15^, ^16^, ^17^. However Dkk3 expression has not been well characterized in human OA tissue nor has its role in chondrocyte biology been explored. Our aim was to assess whether Dkk3 shows aberrant expression in human OA and to establish whether it can regulate chondrocyte behaviour and OA-associated cartilage degradation *in vitro*.

## Materials and methods

### Primary tissue

Primary human OA cartilage and synovium were obtained from age-matched individuals undergoing hip replacement for OA and control cartilage and synovium obtained upon hip replacement for neck-of-femur fracture (NOF); cartilage OA *n* = 13, NOF *n* = 12, OA synovium *n* = 8; NOF synovium *n* = 11. Anteromedial OA (AMG) specimens were obtained from patients undergoing unicompartmental knee replacement (UKR) for OA. Primary human chondrocytes (HAC) were obtained from macroscopically normal regions of the tibial plateau of OA patients undergoing total knee replacement (TKR) and collagenase digested following standard protocols. Explants of cartilage were used for proteoglycan and collagen release assays (DMMB and hydroxyproline respectively). Synovial fluid was collected from individuals undergoing TKR (*n* = 3), UKR (*n* = 3), arthroscopy for cartilage lesions (*n* = 5), matrix-induced autologous chondrocyte implantation (MACI, *n* = 7) or control patients (*n* = 3) with no cartilage lesion but meniscal tears.

Ethical approval (09/H0606/11 and 2005ORTHO7L) was granted by Oxfordshire Research Ethics Committee and East Norfolk and Waveney Research Governance Committee. Informed consent was obtained from all patients.

### Cell culture

SW1353 chondrosarcoma cells (ATCC) and primary HAC were cultured in Dulbecco's Modified Eagle's Medium (DMEM) + 10% (v/v) foetal calf serum (FCS). ATDC5 cells were cultured in DMEM/F12 (Lonza, UK) containing 5% (v/v) FCS, 2 mM glutamine, 10 ug/ml apotransferrin (Sigma) and 30 nM sodium selenite. Confluent ATDC5 cells were stimulated to undergo chondrogenesis by addition of 10 ug/ml insulin (Sigma). Human mesenchymal stem cells (MSCs) (Lonza) were expanded in MSC Growth Medium (Lonza) supplemented with 5 ng/ml fibroblast growth factor-2 (R&D Systems) before high density transwell culture as described^18^, ^19^. Micromass cultures were established as described^20^ before treatment with 100 ng/ml Wnt3a for 4 days.

### Cartilage explant assays

Bovine nasal septum and human articular cartilage were dissected and 2 mm cartilage discs explanted and equilibrated for 24 h before treatment with interleukin-1β (IL1β) (0.5 ng/ml), oncostatin-M (OSM) (5 ng/ml) plus Dkk3 (50, 125 and 250 ng/ml). Treatments were refreshed every 2–3 days and collected forglycosaminoglycan (GAG) and collagen release assays using dimethylmethylene blue (DMMB) and hydroxyproline assays respectively. Remaining cartilage was harvested at 14 days for papain digestion and DMMB and hydroxyproline assays^21^. Control and IL1/OSM-treated explants were collected throughout the time course for RNA extraction (Trizol, Invitrogen, UK), subsequent cDNA synthesis (Superscript, Invitrogen UK) according to manufacturer's instructions prior to quantitative real time PCR (qRT-PCR). Three intra-experimental replicates were carried out for each treatment condition.

### Hip avulsion assay

The hip joint from 5 to 6 week old C57BL/6J mice was dislocated at the femur and the femoral cap avulsed using forceps as previously described^22^. Hip joint cartilage was cultured for 1–48 h in serum-free medium before RNA extraction using Trizol (Invitrogen, UK). cDNA synthesis using Superscript (Invitrogen, UK) was performed prior to qRT-PCR.

### Immunohistochemistry (IHC)

Specimens were fixed in 10% (v/v) formalin for 12 h before decalcification in 5 M HNO_3_, paraffin embedding and cutting into 5 μM sections. Following deparaffinisation and antigen retrieval with 0.2% (v/v) Triton-X 100, sections were blocked and incubated at 4°C overnight in primary antibody (DKK3, R&D Systems, Abingdon, UK) before visualisation using Vectastain ABC (Vector laboratories) with Diaminobenzidine (DAB) and Haematoxylin QS (Vector laboratories).

### Enzyme-linked immunosorbent assay (ELISA)

Dkk3 level in synovial fluid was measured using Dkk3 ELISA (R&D Systems, UK) according to manufacturer's instructions.

### Cytokine treatments

Cells were serum starved overnight and treated with recombinant IL1β (5 ng/ml) and/or OSM (10 ng/ml) for 24 h or pre-treated for 1 h with recombinant Dkk3 (250 ng/ml unless otherwise stated) or carrier alone before addition of recombinant Wnt3a (100 ng/ml, 10 h), activin (20 ng/ml, 6 h) or TGFβ1 (4 ng/ml, 6 h). All cytokines from R&D Systems. At least three intra-experimental replicates were carried out per cytokine treatment.

Following cytokine treatment cDNA was synthesized using MMLV from DNase-treated cell lysates harvested in Cells-to-cDNA lysis buffer (Ambion) according to manufacturer's instructions.

### qRT-PCR

Expression of genes was measured by qRT-PCR on a ViiA7 (Applied Biosystems). Relative quantification is expressed as $2^{-\Delta C_{\text{t}}}$, where Δ*C*_t_ is *C*_t_(gene of interest) − *C*_t_(18S rRNA). Samples which gave a *C*_t_ reading + 1.5*C*_t_ greater or less than the median for *18S* were excluded from further analyses.

### Luciferase assays

SW1353 chondrosarcoma cells were used for plasmid transfections using Lipofectamine 2000 with the Smad-responsive reporter (CAGA)_12_-luc, Wnt-responsive 8xTCF/LEF binding site (TOPFlash) and mutant TCF/LEF site control FOPFlash and β-galactosidase transfection control plasmid^23^, ^24^. Cells were treated with Wnt3a (100 ng/ml) for 10 h or TGFβ (4 ng/ml) or activin (20 ng/ml) for 3 h with and without 1 h Dkk3 pre-incubation before measurement of luciferase activity using the Luciferase and Beta-Glo assay systems (Promega).

### small interfering RNA (siRNA)

Cells (HAC and SW1353) were transfected with 2.5 nM of siRNA against Dkk3 (Qiagen, siDkk3) or Allstars non-targeting negative control (Qiagen, siNegative) using Dharmafect (Thermoscientific, UK) according to manufacturer's instructions. Cells were transfected 48 h prior to cytokine treatment.

### Statistical analysis

Analyses were carried out using Graphpad Prism 6.0. Student's *t* test was used to test differences between two samples whilst analysis of variance (ANOVA) with either Dunnett's or Tukey post-test was used for multiple samples. Normality was tested using the Shapiro–Wilk test. *P* < 0.05 was considered statistically significant. *≤0.05, **≤0.01, ***≤0.001. Graphs show mean ± 95% confidence intervals of biological (patient or cell) replicates.

## Results

### Dkk3 expression is upregulated in OA tissue

**Fig. 1:**
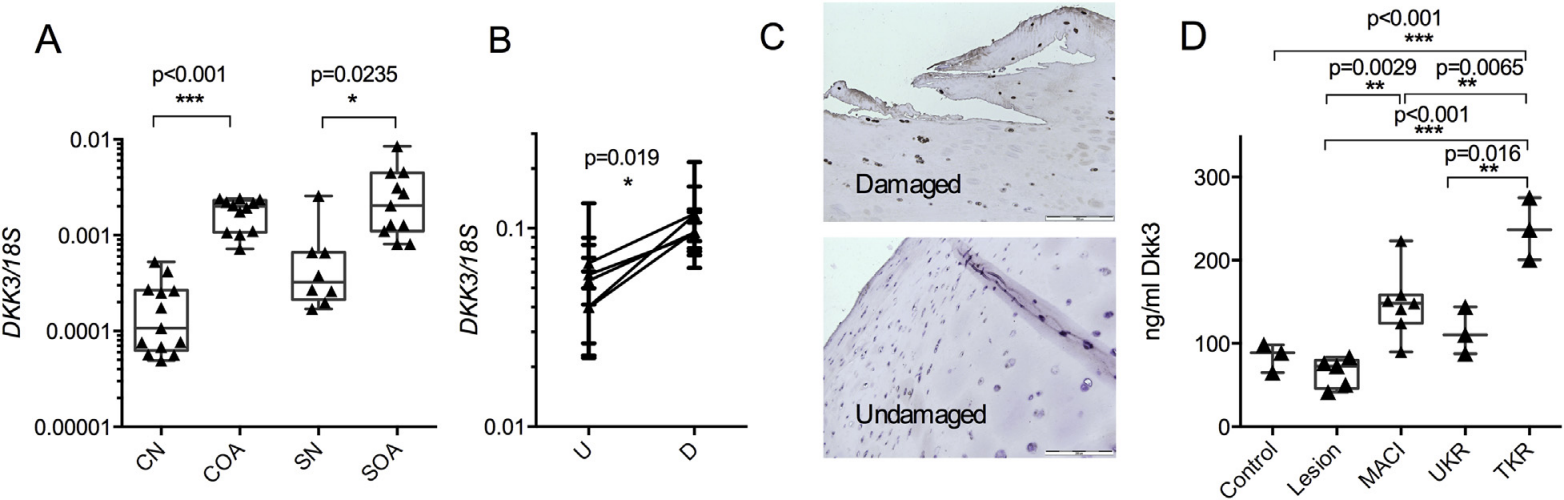
Dkk3 levels are altered in OA. (A) *DKK3* expression is elevated in OA cartilage and synovium from patients undergoing total hip arthroplasty. OA cartilage = COA, *n* = 13, NOF control cartilage (CN, *n* = 11), OA synovium (SOA, *n* = 8) and NOF control synovium (SN, *n* = 11). Dkk3 gene (B) and protein (C) levels were elevated in damaged compared to undamaged cartilage from individuals with AMG (*n* = 5), IHC scale bar = 20 μM. (D) Dkk3 protein measured by ELISA of synovial fluid was increased in individuals undergoing TKR for OA, *n* = 3. Levels were also measured in individuals with no cartilage lesions (control, *n* = 3), undergoing arthroplasty for cartilage lesions (lesion, *n* = 5), MACI (*n* = 7) following arthroscopy, or UKR (*n* = 3) for AMG. (A, B) analysed by *t* test, (D) by ANOVA with Tukey post-test, three technical replicates per patient with the mean of these used in statistical analysis and represented as a dot (biological replicate) on each graph.

Expression of *DKK3* mRNA was increased >10-fold (*P* < 0.0001) in OA cartilage compared to NOF control [Fig. 1(A)]. Analysis of synovium from OA patients and NOF controls showed a 3.2-fold (*P* = 0.0235) increase in *DKK3* mRNA in diseased tissue. *DKK3* mRNA expression [Fig. 1(B)] was 2.1-fold (*P* = 0.019) higher in damaged cartilage from patients with AMG. Our previous work shows reduced matrix metalloproteinase (*MMP*) and mRNA expression in damaged compared to undamaged cartilage^25^. Immunohistochemistry in AMG patients also showed significant Dkk3 staining in the superficial zone of damaged but not undamaged cartilage [Fig. 1(C)]. Dkk3 protein [Fig. 1(D)] in synovial fluid was 2.1-fold higher (*P* = 0.0002) in patients undergoing TKR for OA compared to control individuals, those with cartilage lesions (4.33-fold, *P* < 0.0001) or patients undergoing UKR (2.83-fold, *P* = 0.0016). Matrix-induced autologous chondrocyte implantation (MACI) is performed 4–6 weeks following initial assessment of cartilage lesions by arthroscopy. Dkk3 levels at the time of MACI were significantly higher than at arthroscopy (i.e., lesion) (2.3-fold, *P* = 0.0029).

### *DKK3* expression is downregulated following cartilage injury and during chondrogenesis

**Fig. 2:**
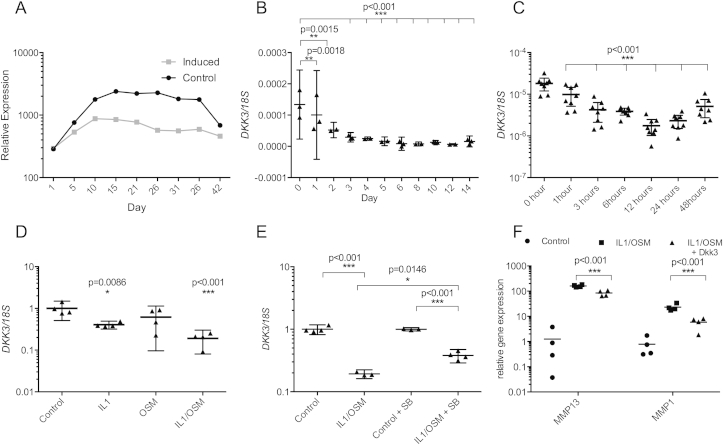
Dkk3 is regulated by inflammatory cytokines and injury and during chondrogenesis. Dkk3 gene expression was reduced during chondrogenesis of ATDC5 cells (microarray) (A) and human MSCs (qRT-PCR, n = 2–3 biological replicates) (B). (C) qRT-PCR of RNA extracted from murine hip cartilage following *ex vivo* avulsion showed a reduction in *Dkk3* expression (*n* = 8 mice). (D) 24 h treatment with IL1β and IL1β/OSM reduced *DKK3* expression in primary monolayer HAC (*n* = 4 patients, four technical replicates per condition), this was partially inhibited by 10 μM of the p38 mitogen activated protein kinase (MAPK) inhibitor SB202190 (SB) (*n* = 4 patients, four technical replicates per condition) (E). (F) IL1/OSM-induced *MMP13* and *MMP1* expression was inhibited by Dkk3 (*n* = 4 patients, four technical replicates per condition). (BF) ANOVA with Dunnett's post-test. All statistical analysis carried out on biological replicates.

The OA phenotype includes reinitiation of development^26^, thus establishing Dkk3 regulation in chondrogenesis is important. ATDC5 differentiation is an established model of chondrogenesis. Following chondrogenic differentiation, microarray analysis showed *Dkk3* expression decreased relative to non-induced control cultures [Fig. 2(A)]. Expression of chondrogenic markers collagen, type II, alpha I (Col2a1) and aggrecan (*Acan*) (data not shown) were increased across these time points^23^. Human MSCs in high density transwell cultures also showed a significant 1.3–21-fold reduction (*P* < 0.01) in *DKK3* expression throughout chondrogenic differentiation into cartilage discs [Fig. 2(B)], with increases in *COL2A1* and *ACAN* across the time course^18^.

Joint injury is associated with secondary OA therefore Dkk3 regulation during injury or in response to inflammatory mediators of injury was investigated. *Dkk3* expression in murine cartilage was decreased 1.8-fold (*P* = 0.0005) immediately (1 h) following hip avulsion injury and remained low (3.54-fold reduction, *P* < 0.0001) 48 h after injury [Fig. 2(C)]. Treatment of HAC for 72 h with IL1β or the combination IL1β/OSM reduced *DKK3* expression (2.4-fold, *P* = 0.0086 and 5.25-fold, *P* = 0.0009) [Fig. 2(D)], this was partially inhibited by inhibition of p38 MAPK activity [Fig. 2(E)]. IL1β/OSM treatment of HAC induced *MMP13* and *MMP1* expression [Fig. 2(F)], this was inhibited by Dkk3 (1.9-fold, *P* < 0.0001 and 3.9-fold, *P* < 0.0001), suggesting Dkk3 inhibits IL1/OSM-induced cartilage degradation via modulation of MMP levels.

### Dkk3 prevents cartilage degradation *in vitro*

**Fig. 3:**
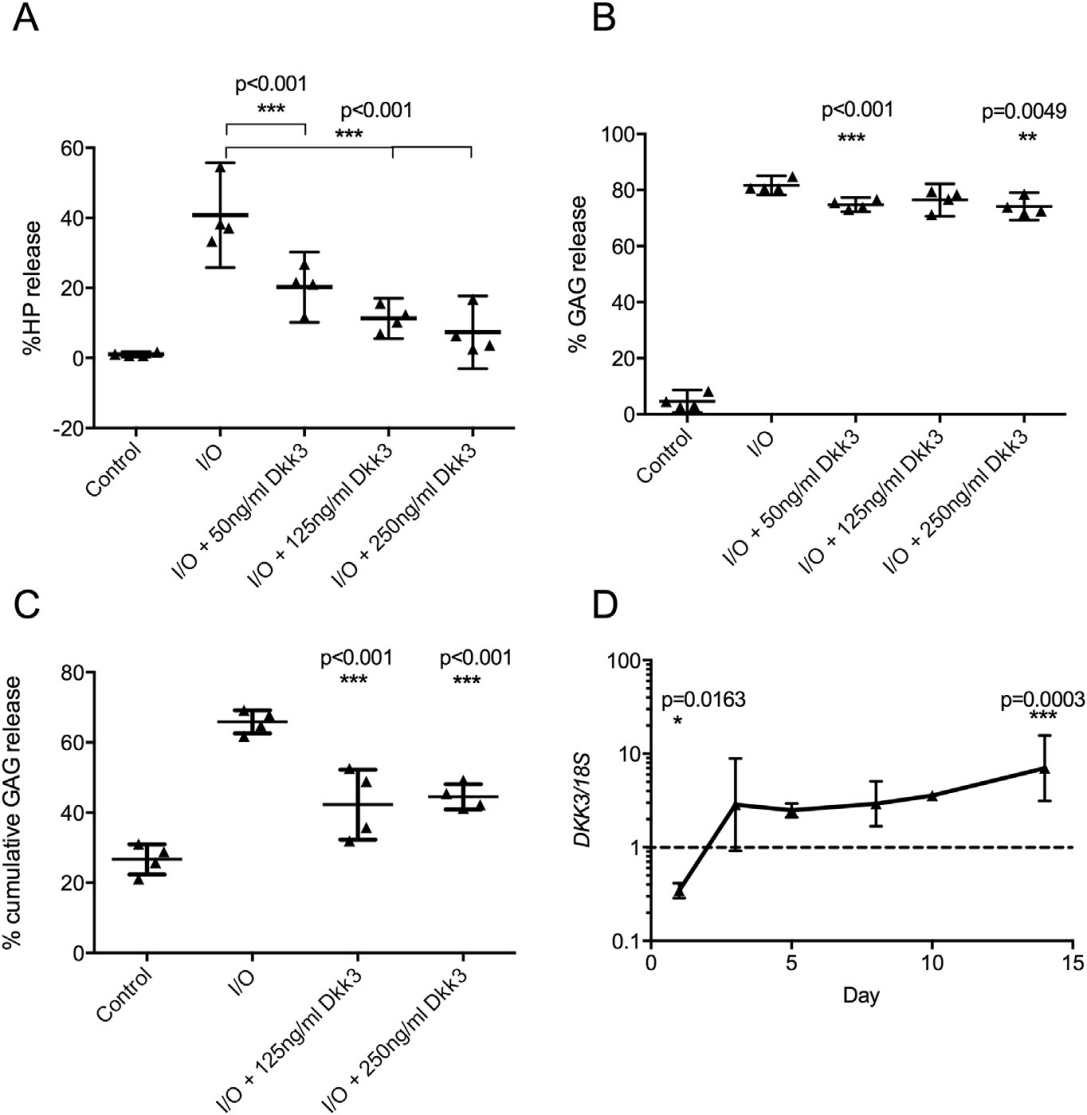
Dkk3 inhibits *ex vivo* cartilage degradation. (A) Dkk3 reduced IL1/OSM-induced collagen degradation (hydroxyproline release) from BNC explants (*n* = 4 biological replicates, three technical replicates per condition). (B) BNC (*n* = 4) and (C) human knee (*n* = 4) cartilage explants showed a reduction in proteoglycan degradation (GAG release, DMMB assay) in the presence of Dkk3 compared to IL1/OSM treatment alone, three technical replicates per condition. (D) *DKK3* expression was significantly reduced in BNC (*n* = 3) at day 1 of IL1/OSM treatment and increased from day 5 onwards. (A), (B) and (C) ANOVA with Dunnett's post-test relative to IL1/OSM alone (D) *t* test relative to untreated timepoint control. I/O = IL1/OSM. All statistical analysis carried out on biological replicates (each biological replicate the mean of technical replicates for that sample).

OA is characterized by loss of proteoglycan and collagen from cartilage ECM. Bovine nasal cartilage (BNC) explants were treated with IL1β/OSM ± recombinant Dkk3. Cytokine-induced collagen loss [Fig. 3(A)] at day 14 was dose-dependently inhibited by addition of 50, 125 or 250 ng/ml Dkk3 (2.0-, 3.6- and 5.6-fold reduction, *P* < 0.001) IL1β/OSM-induced proteoglycan loss from BNC explants was also dose-dependently inhibited by 250 ng/ml Dkk3 [1.1-fold, *P* = 0.0049, Fig. 3(B)]. Human explants cannot be induced to release collagen however they showed [Fig. 3(C)] significant dose-dependent inhibition of cytokine-induced proteoglycan loss in the presence of 125 ng/ml and 250 ng/ml Dkk3 (1.6- and 1.5-fold, *P* = 0.003 and *P* = 0.0008, respectively). *DKK3* expression was decreased 1 day after IL1/OSM treatment of BNC explants before increased expression from day 3 onwards [Fig. 3(D)]. No toxicity was detected (lactate dehydrogenase assay) during 14 days treatment with Dkk3 (data not shown).

### Dkk3 inhibits Wnt signaling

**Fig. 4:**
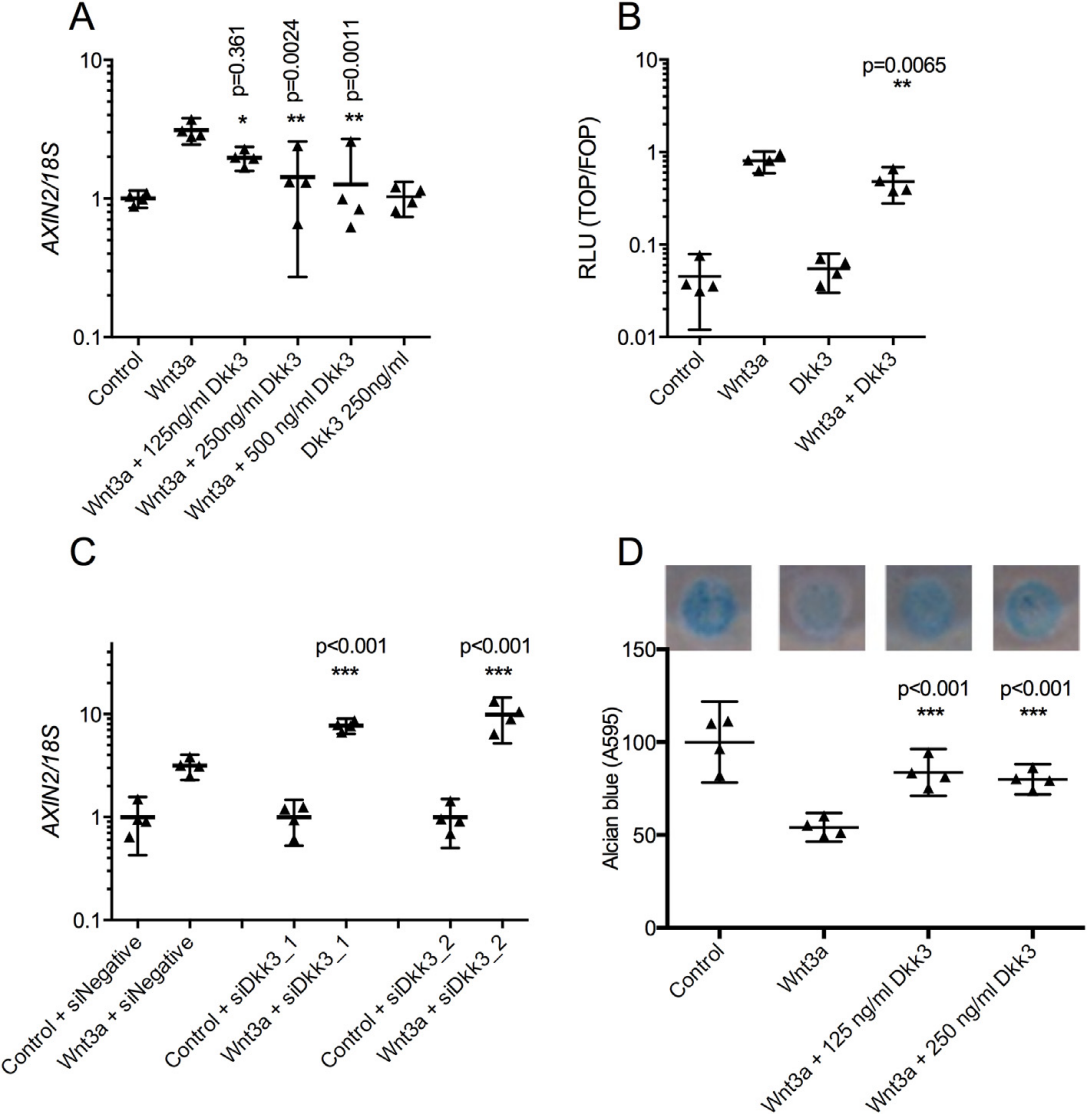
Dkk3 inhibits Wnt signaling in chondrocytes. (A) HAC (*n* = 4 patients, three technical replicates per condition) were treated with Wnt3a with 0–500 ng/ml Dkk3 and *AXIN2* expression was reduced in the presence of Dkk3. (B) SW1353 cells were transfected with the TOPFlash reporter plasmid and FOPFlash control. Luminescence was assessed following treatment with Wnt3a, Dkk3 or the combination of Wnt3a and Dkk3. Dkk3 reduced Wnt3a-induced luciferase activity (*n* = 8). (C) Primary HAC (*n* = 4) were treated with siRNA against Dkk3 or negative control siRNA. In the absence of Dkk3 there was a relative increase in Wnt3a-induced *AXIN2* expression. (D) Dkk3 inhibited the Wnt3a-induced reduction in proteoglycan production of HAC grown in micromass culture (n = 4) as measured by alcian blue staining, mean ± SD. ANOVA with Dunnett's post-test, (A, B, D) significance shown for comparisons of Wnt3a to Wnt3a + Dkk3, (C) significance shown for comparisons of Control + siDkk3 to Wnt3a + SiDkk3. *n* represents biological replicates (the mean of three technical replicates per condition for luciferase assays and four technical replicates per condition for gene expression assays). All statistical analysis carried out on biological replicates.

Dkk3 is a non-canonical member of the Dkk family of Wnt antagonists with tissue-dependent effects on Wnt signaling activity. To determine whether Dkk3 did regulate Wnt signaling in cartilage we treated HAC with Dkk3 and Wnt3a. The Wnt3a-induced increase of the Wnt target gene axis inhibition protein 2 (*AXIN2*) [Fig. 4(A)] was decreased in HAC by co-incubation with Wnt3a and 125, 250 or 500 ng/ml Dkk3 (1.6-, 2.2- and 2.5-fold, *P* = 0.0050, <0.0001, <0.0001 respectively) compared to Wnt3a alone. Furthermore the activity of the Wnt-responsive TOPFlash reporter was reduced by the addition of Dkk3 (1.7-fold, *P* = 0.0010) [Fig. 4(B)] compared to Wnt3a alone. Knockdown of Dkk3 in HAC increased Wnt3a-induced *AXIN2* expression compared to a non-targeting siRNA control [Fig. 4(C)]. Micromass cultures of HAC show significant reduction in proteoglycan production following Wnt3a treatment for 4 days [Fig. 4(D)]. Proteoglycan levels were restored by addition of Dkk3 demonstrating inhibition of Wnt3a-mediated effects on proteoglycan synthesis.

### Dkk3 regulates TGFβ signaling

**Supplementary Fig. 1:**
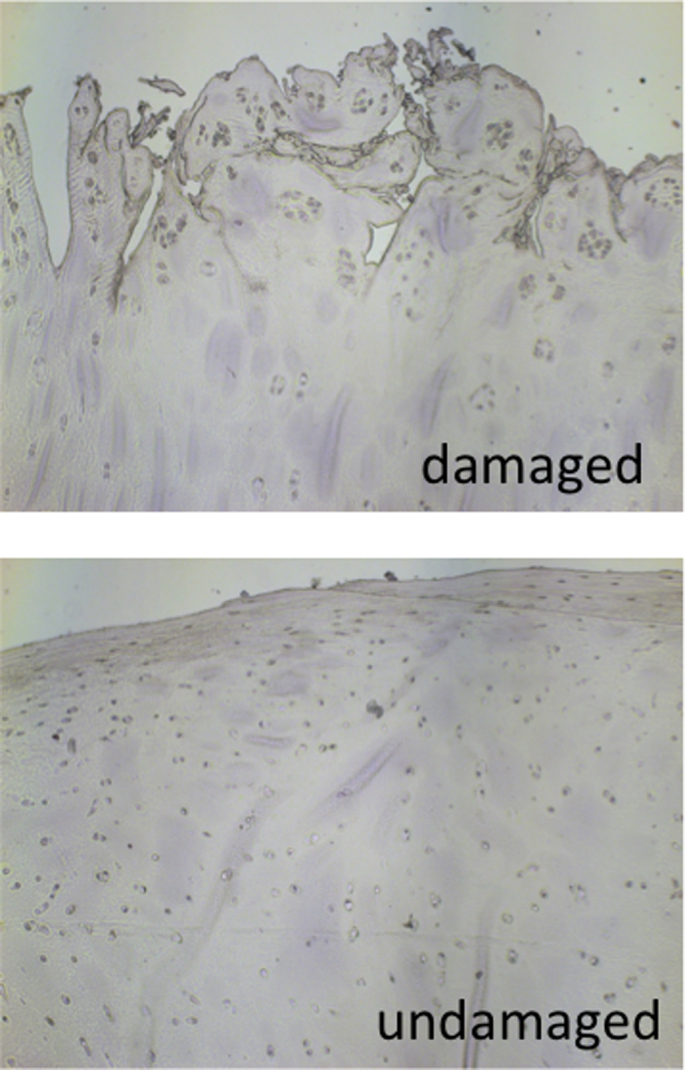
Isotype IgG control on specimens of damaged and undamaged cartilage from AMG patients.

**Supplementary Fig. 2:**
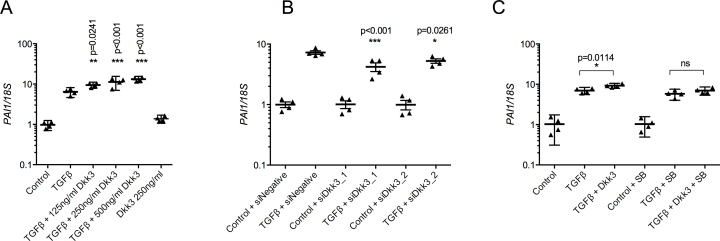
Dkk3 enhances TGFβ-induced *PAI1* expression. (A) HAC (*n* = 4 patients) showed increased *PAI1* expression in the presence of Dkk3 plus TGFβ compared to treatment with TGFβ alone. (B) Treatment of HAC (*n* = 4 patients) with siRNA against Dkk3 reduced TGFβ-induced *PAI1* expression compared to HAC treated with non-targeting negative control siRNA. (C) Inhibition of p38 MAPK signaling in HAC (*n* = 4 patients) with SB202190 (SB) removed the Dkk3-induced enhancement of *PAI1* expression following TGFβ treatment. (A, B) ANOVA with Dunnett's post-test. (C) ANOVA with Tukey post-test. *n* = number of biological replicates, four technical replicates were carried out and the mean calculated to generate the value used for each biological replicate. All statistical analysis carried out on biological replicates.

**Fig. 5:**
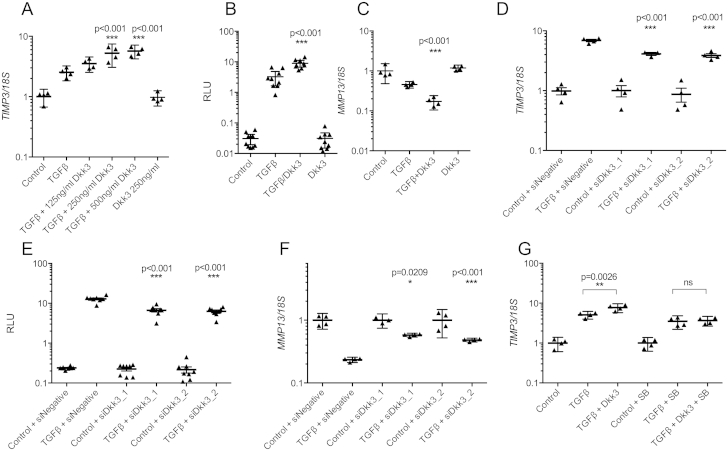
Dkk3 enhances TGFβ signaling response. (A) HAC (*n* = 4) treated with TGFβ showed increased *TIMP3* expression in the presence Dkk3 compared to TGFβ alone. (B) TGFβ-responsive (CAGA)_12_-luciferase activity in SW1353 cells (*n* = 8) was also enhanced by Dkk3 compared to TGFβ alone. (C) Dkk3 treatment decreased *MMP13* expression in HAC compared to TGFβ treatment alone (*n* = 4). TGFβ-induced *TIMP3* expression (D, *n* = 4) and (CAGA)_12_-luciferase activity (E, *n* = 8) was reduced following knockdown of Dkk3. (F) siRNA against Dkk3 partially inhibited the TGFβ-induced reduction in *MMP13* expression in HAC (*n* = 4). (G) Inhibition of HAC p38 MAPK activity by treatment with 10 μM SB202190 (SB) abolished the Dkk3-induced enhancement of *TIMP3* expression following TGFβ treatment (*n* = 3). (A–F) ANOVA with Dunnett's post-test, significance shown for comparison between TGFβ alone and TGFβ + Dkk3 (A–C) and for TGFβ + siRNA to control + siRNA (D–F). (G) ANOVA plus Tukey post-test, significance shown for comparison of TGFβ + Dkk3 to TGFβ alone for with and without SB202190. *n* represents biological replicates (the mean of three technical replicates per condition for luciferase assays and four technical replicates per condition for gene expression assays). All statistical analysis carried out on biological replicates.

TGFβ signaling responsiveness is reduced in ageing and OA. Expression of the TGFβ-responsive gene, tissue inhibitor or metalloproteinase-3 (*TIMP3*) ^27^, was dose-dependently enhanced in HAC treated with TGFβ plus 250 and 500 ng/ml Dkk3 compared to TGFβ alone (2.1- and 2.2-fold, *P* < 0.001) [Fig. 5(A)]. TGFβ-responsive plasminogen activator inhibitor-1 (*PAI1*) [Supplementary Fig. 2(A)] a disintegrin and metalloproteinase-12 (*ADAM12*) (data not shown) were also enhanced whilst *MMP13* expression was decreased by TGFβ in combination with 250 ng/ml Dkk3 [Fig. 5(C)] compared to TGFβ alone (2.6-fold, *P* < 0.001). 250 ng/ml Dkk3 also increased activity of the TGFβ-responsive (CAGA)_12_-luciferase reporter in SW1353 cells relative to TGFβ alone (2.8-fold, *P* < 0.0001) [Fig. 5(B)]. No effect of Dkk3 alone was seen on *TIMP3*, *PAI1* or *ADAM12* gene expression or CAGA-luc induction. The extent of TGFβ induction of *TIMP3* [Fig. 5(D)], *PAI1* [Supplementary Fig. 1(B)] and *ADAM12* (data not shown) expression and CAGA-luc [Fig. 5(E)] activity was decreased by Dkk3 knockdown. Knockdown of Dkk3 partially repressed the TGFβ-induced decrease of *MMP13* in primary HAC [Fig. 5(F)]. p38 MAPK-mediated stabilization of Smad4 has been described in *Xenopus laevis*^28^, therefore we inhibited p38 MAPK. The induction of TGFβ-induced *TIMP3* [Fig. 5(G)] and *PAI1* [Supplementary Fig. 2(B)] expression by Dkk3 was abrogated following p38 inhibition in HAC [Fig. 5(G)].

**Supplementary Fig. 3:**
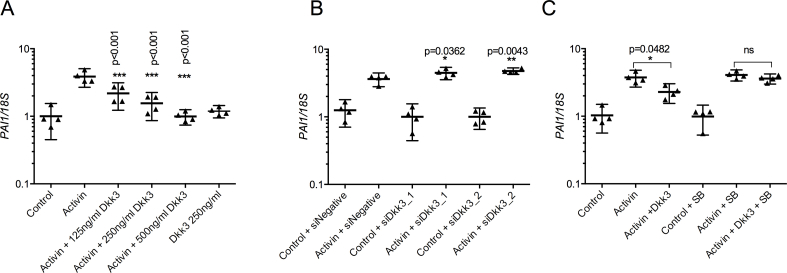
Dkk3 inhibits Activin-induced *PAI1* expression. (A) HAC (*n* = 4 patients) showed decreased *PAI1* expression in the presence of Dkk3 plus Activin compared to treatment with Activin alone. (B) Treatment of HAC (*n* = 4 patients) with siRNA against Dkk3 increased Activin-induced *PAI1* expression compared to HAC treated with non-targeting negative control siRNA. (C) Inhibition of p38 MAPK signaling in HAC (*n* = 4 patients) with SB202190 (SB) removed the Dkk3-induced reduction of *PAI1* expression following Activin treatment. (A, B) ANOVA with Dunnett's post-test. (C) ANOVA with Tukey post-test. *n* = number of biological replicates, four technical replicates were carried out and the mean calculated to generate the value used for each biological replicate. All statistical analysis carried out on biological replicates.

**Fig. 6:**
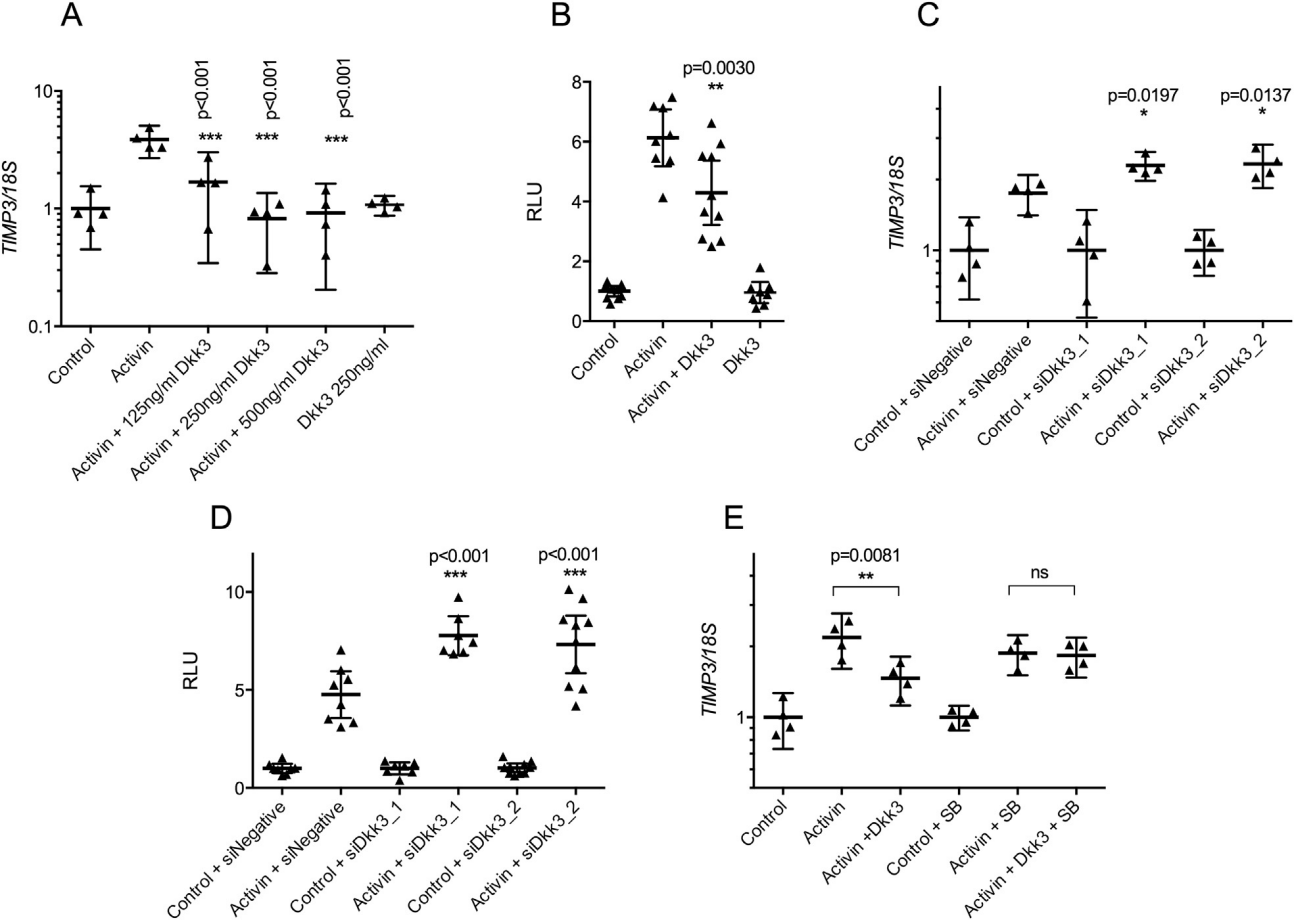
Dkk3 inhibits activin signaling response. (A) HAC (*n* = 4) treated with activin showed decreased *TIMP3* expression in the presence Dkk3 compared to Activin alone. (B) (CAGA)_12_-luciferase activity in SW1353 cells (*n* = 8) was also reduced in the presence of Dkk3 compared to activin alone. Activin-induced *TIMP3* expression (C, *n* = 4) and (CAGA)_12_-luciferase activity (D, *n* = 4) was increased following knockdown of Dkk3. (E) Inhibition of HAC p38 MAPK activity by treatment with 10 μM SB202190 (SB) abolished the Dkk3 (250 ng/ml)-induced reduction in *TIMP3* expression following Activin treatment (*n* = 4). (A–D) ANOVA with Dunnett's post-test, significance shown for comparison between Activin and Activin + Dkk3 (A, B) and between Activin + siDkk3 to Control + siDkk3 (C, D). (E) ANOVA with Tukey post-test, significance shown for comparison between Activin alone and Activin + Dkk3 in the absence and presence of SB202190. *n* represents biological replicates (the mean of three technical replicates per condition for luciferase assays and four technical replicates per condition for gene expression assays). All statistical analysis carried out on biological replicates.

Activin is a member of the TGFβ superfamily that also signals via Smad2/3. To assess whether Dkk3 impacted other Smad2/3-related signaling pathways, HAC and SW1353 were treated with activin ± Dkk3. Activin-induced *TIMP3* expression and (CAGA)_12_-luc activity whilst co-incubation with Dkk3 caused a dose-dependent reduction in both of these outputs [Fig. 6(A and B)]. Knockdown of Dkk3 enhanced activin-induced *TIMP3* expression and CAGA-luc activity suggesting endogenous Dkk3 may act to reduce cellular activin-induced responses [Fig. 6(C and D)]. There was no repression of HAC *TIMP3* expression when p38 MAPK activity was inhibited [Fig. 6(E)]. Activin-induced *PAI1* expression followed the same trends as *TIMP3* [Supplementary Fig. 3(A–C)].

## Discussion

Altered expression of cytokines and consequent disruption of cell signaling is associated with OA pathogenesis. Dkk3 is a non-canonical member of the Dkk family of Wnt antagonists that has not been explored in cartilage biology despite numerous studies noting its increased expression in models of OA. In this study we demonstrate that Dkk3 is upregulated in adult human OA cartilage and synovial tissue but is decreased during chondrogenesis. Dkk3 protects against *in vitro* cartilage degradation and its expression is regulated by both injury and inflammatory cytokines. Wnt and activin signaling are both inhibited by Dkk3 whilst TGFβ signaling is enhanced. The upregulation of Dkk3 in OA may be a protective mechanism to limit cartilage damage and to regulate aberrant cell signaling associated with disease.

OA is a complex disease affecting multiple joint tissues, with a unique combination of factors likely to regulate pathogenesis within each tissue and across different joint locations. We show that Dkk3 is upregulated in both hip and knee OA and in both synovial tissue and cartilage from diseased joints. Dkk3 upregulation is also reported in OA subchondral bone from patients undergoing TKR^29^. This suggests Dkk3 is relevant to whole joint biology in two common sites of disease. The increased Dkk3 in synovial fluid of patients with tricompartmental OA may implicate Dkk3 as a biomarker distinguishing end-stage disease. Further studies of Dkk3 as a circulating biomarker are warranted.

Dysregulation of Wnt and TGFβ family members has been strongly implicated in experimental and human OA^5^, ^6^. An imbalance in Wnt signaling leads to OA development in murine models, and Wnt antagonists *DKK1* and *FRZB* have been reported as downregulated in human OA^30^, ^31^, ^32^. Wnts and activin are also released following cartilage injury^33^, ^34^. TGFβ signaling and responsiveness decrease with age and OA development, whilst increased activin has been detected in OA tissues^34^, ^35^. Dkk3 has both agonistic and antagonistic effects on the Wnt pathway dependent on tissue of expression and thus investigation of its impact on Wnt signaling in cartilage was investigated in our study^7^, ^8^, ^9^. Opposing regulatory roles of Dkk3 on TGFβ signaling in *Xenopus* and prostate cancer^13^, ^28^ have been reported but its function in musculoskeletal tissue has not been studied.

In adult HAC we have shown that Dkk3 antagonized Wnt signaling and protected against Wnt-induced proteoglycan reduction. Dkk3 enhanced TGFβ signaling in chondrocytes and interestingly was necessary for TGFβ-induced reduction of *MMP13* expression. Dkk3 may mediate protective effects on cartilage partially through upregulation of TGFβ signaling and inhibition of Wnt signaling. Surprisingly, Dkk3 inhibited activin signaling in cartilage despite both activin and TGFβ commonly signaling through Smad2/3. Inhibition of p38 MAPK signaling abrogated the effects of Dkk3 on both TGFβ and activin signaling which shows Dkk3 action here is p38 MAPK dependent. A previous study demonstrated Dkk3-dependent Smad4-stabilization by p38 MAPK and this requires further investigation in chondrocytes^36^. Our data may indicate that Dkk3 effects on TGFβ require p38 MAPK for stabilization of Smad4. The effect of Dkk3 on activin signaling is also p38 MAPK dependent but may operate through a pathway that does not use Smad4. The mechanism by which differential regulation of activin and TGFβ can occur is currently unknown and beyond the scope of this study.

Injury to the joint commonly leads to OA development. To model cartilage injury *ex vivo* the murine hip was avulsed and Dkk3 levels found to be decreased within 1 h. Decreased Dkk3 protein was also shown in pilot data from an *ex vivo* porcine explant model^37^ following cutting injury (data not shown). Treatment with IL1β/OSM in our study led to a reduction in *DKK3* expression that was partially p38 MAPK dependent. In contrast, previous reports on murine OA^15^, ^16^, ^17^ and our data in human tissue show an increase in Dkk3 expression in established disease. Dkk3 may be regulated in a temporal manner during disease pathogenesis. This is supported by our BNC data that shows an initial decrease in *DKK3* expression followed by an increase as cartilage degradation occurs. It is also of note that levels of Dkk3 protein in synovial fluid were lower at the time of arthroscopy than 4–6 weeks later when MACI was performed. This may indicate that injury to the joint capsule leads to significant Dkk3 release from other joint tissues that overcomes any decrease due to cartilage injury. The sources of Dkk3 in the joint require further investigation. The initial injury response leading to decreased Dkk3 may be completed at MACI with Dkk3 levels consequently increased in the ensuing, later stage repair attempt.

Paralleling the potential roles of the Wnt and TGFβ pathways in OA pathogenesis, chondrogenesis and articular cartilage development require TGFβ signaling as well as regulation of Wnt signaling^5^, ^38^. Given the reversion of OA chondrocytes to a developmental-like phenotype^39^ our data showing decreased *DKK3* during chondrogenesis, shows a potential role for Dkk3 in chondrogenesis, and also suggests that the immediate downregulation of *DKK3* in injury may be an early repair response.

Strikingly, Dkk3 protected against IL1β/OSM-stimulated cartilage degradation. The increase in Dkk3 in OA may be a protective mechanism to minimize cartilage degradation and the OA-associated shift in chondrocyte phenotype. This is supported by the reduction in cartilage-degrading *MMP13* expression by Dkk3 in the presence of IL1β/OSM. Microarray analysis of HAC treated with siRNA against Dkk3 did not reveal pathways of Dkk3 action on unstimulated cells (data not shown), thus future analysis will use cytokine-stimulated cells. However siRNA treatment did increase *MMP13* expression in TGFβ-treated cells suggesting that Dkk3 may limit cartilage damage partially through reduction of both IL1β/OSM and TGFβ-effects on *MMP13*.

Overall Dkk3 upregulation in disease may be a defence mechanism to counteract disease-related dysregulation of cell signaling pathways; inhibiting inflammatory cytokine effects on cartilage degradation and enhancing TGFβ signaling whilst maintaining regulation of Wnt signaling in an attempt to counteract disease-associated changes in these pathways. Supplementation with Dkk3 at an early stage of disease or post-injury may therefore be therapeutically beneficial.

Further investigation of Dkk3 in murine models of OA is necessary to ascertain its contribution to cartilage homeostasis and disease pathogenesis. Although the Dkk3 null mouse^40^does not have an overt musculoskeletal phenotype our preliminary analysis suggests increased knee OA in 6-month old animals, we are currently investigating injury-models of OA. Dkk3 gene therapy is in clinical trial for prostate cancer with promising results^41^, but further preclinical evaluation is necessary alongside more detailed investigation of the role of Dkk3 in other tissues of the healthy and OA joint.

In summary we have demonstrated that Dkk3 is upregulated in human OA and reduces cartilage degradation. These findings may have clinical implications as treatment with Dkk3 may prevent cartilage degeneration in OA and early intervention with Dkk3-based therapy may slow OA progression.

## Contributors

SJBS and IMC designed the study. SJBS, RKD, TES, MJB, KC and LL carried out data acquisition. AJC and AP provided patient samples and assisted with data interpretation. SJBS and IMC carried out data analysis and interpretation. All authors helped prepared the manuscript and approved the manuscript for submission.

## Conflict of interests

The authors have no competing interests to declare.

## Funding

Grants: Arthritis Research UK grants 20087 (SJBS), 19222 (SJBS), 19424 (MJB) and the National Institute of Health Research (NIHR) Oxford Musculoskeletal Biomedical Research Unit funded this work.

## References

[1] Wojdasiewicz P., Poniatowski L.A., Szukiewicz D. (2014). The role of inflammatory and anti-inflammatory
cytokines in the pathogenesis of osteoarthritis. Mediators Inflamm.

[2] Andriacchi T.P., Favre J. (2014). The nature of in vivo mechanical signals that
influence cartilage health and progression to knee
osteoarthritis. Curr Rheumatol Rep.

[3] Loeser R.F. (2013). Aging processes and the development of
osteoarthritis. Curr Opin Rheumatol.

[4] Goldring M.B. (2000). The role of the chondrocyte in
osteoarthritis. Arthritis Rheum.

[5] Staines K.A., Macrae V.E., Farquharson C. (2012). Cartilage development and degeneration: a Wnt Wnt
situation. Cell Biochem Funct.

[6] van der Kraan P.M., Goumans M.J., Blaney Davidson E., ten Dijke P. (2012). Age-dependent alteration of TGF-beta signalling in
osteoarthritis. Cell Tissue Res.

[7] Nakamura R.E., Hunter D.D., Yi H., Brunken W.J., Hackam A.S. (2007). Identification of two novel activities of the Wnt
signaling regulator Dickkopf 3 and characterization of its
expression in the mouse retina. BMC Cell Biol.

[8] Ueno K., Hirata H., Majid S., Chen Y., Zaman M.S., Tabatabai Z.L. (2011). Wnt antagonist DICKKOPF-3 (Dkk-3) induces apoptosis in
human renal cell carcinoma. Mol Carcinog.

[9] Yue W., Sun Q., Dacic S., Landreneau R.J., Siegfried J.M., Yu J. (2008). Downregulation of Dkk3 activates beta-catenin/TCF-4
signaling in lung cancer. Carcinogenesis.

[10] Wang Z., Ma L.J., Kang Y., Li X., Zhang X.J. (2015). Dickkopf-3 (Dkk3) induces apoptosis in
cisplatin-resistant lung adenocarcinoma cells via the
Wnt/beta-catenin pathway. Oncol Rep.

[11] Kinoshita R., Watanabe M., Huang P., Li S.A., Sakaguchi M., Kumon H. (2015). The cysteine-rich core domain of REIC/Dkk-3 is
critical for its effect on monocyte differentiation and tumor
regression. Oncol Rep.

[12] Meister M., Papatriantafyllou M., Nordstrom V., Kumar V., Ludwig J., Lui K.O. (2015). Dickkopf-3, a tissue-derived modulator of local T-cell
responses. Front Immunol.

[13] Romero D., Kawano Y., Bengoa N., Walker M.M., Maltry N., Niehrs C. (2013). Downregulation of Dickkopf-3 disrupts prostate acinar
morphogenesis through TGF-beta/Smad signalling. J Cell Sci.

[14] Zhang Y., Liu Y., Zhu X.H., Zhang X.D., Jiang D.S., Bian Z.Y. (2014). Dickkopf-3 attenuates pressure overload-induced
cardiac remodelling. Cardiovasc Res.

[15] Blom A.B., Brockbank S.M., van Lent P.L., van Beuningen H.M., Geurts J., Takahashi N. (2009). Involvement of the Wnt signaling pathway in
experimental and human osteoarthritis: prominent role of
Wnt-induced signaling protein 1. Arthritis Rheum.

[16] Meng J., Ma X., Ma D., Xu C. (2005). Microarray analysis of differential gene expression in
temporomandibular joint condylar cartilage after experimentally
induced osteoarthritis. Osteoarthritis Cartilage.

[17] Loeser R.F., Olex A.L., McNulty M.A., Carlson C.S., Callahan M.F., Ferguson C.M. (2012). Microarray analysis reveals age-related differences in
gene expression during the development of osteoarthritis in
mice. Arthritis Rheum.

[18] Barter M.J., Gómez R., Woods S., Hui W., Smith G.R., Shanley D.P. (33, 2015, 3266–3280). Genome-wide microRNA and gene analysis of mesenchymal
stem cell chondrogenesis identifies an essential role and
multiple targets for miR-140-5p. Stem Cells.

[19] Murdoch A.D., Grady L.M., Ablett M.P., Katopodi T., Meadows R.S., Hardingham T.E. (2007). Chondrogenic differentiation of human bone marrow stem
cells in transwell cultures: generation of scaffold-free
cartilage. Stem Cells.

[20] Greco K.V., Iqbal A.J., Rattazzi L., Nalesso G., Moradi-Bidhendi N., Moore A.R. (2011). High density micromass cultures of a human chondrocyte
cell line: a reliable assay system to reveal the modulatory
functions of pharmacological agents. Biochem Pharmacol.

[21] Davidson R.K., Jupp O., de Ferrars R., Kay C.D., Culley K.L., Norton R. (2013). Sulforaphane represses matrix-degrading proteases and
protects cartilage from destruction in vitro and
in vivo. Arthritis Rheum.

[22] Chong K.W., Chanalaris A., Burleigh A., Jin H., Watt F.E., Saklatvala J. (2013). Fibroblast growth factor 2 drives changes in gene
expression following injury to murine cartilage in vitro and
in vivo. Arthritis Rheum.

[23] Swingler T.E., Wheeler G., Carmont V., Elliott H.R., Barter M.J., Abu-Elmagd M. (2012). The expression and function of microRNAs in
chondrogenesis and osteoarthritis. Arthritis Rheum.

[24] Korinek V., Barker N., Morin P.J., van Wichen D., de Weger R., Kinzler K.W. (1997). Constitutive transcriptional activation by a
beta-catenin-Tcf complex in APC^−/−^ colon
carcinoma. Science.

[25] Snelling S., Rout R., Davidson R., Clark I., Carr A., Hulley P.A. (2014). A gene expression study of normal and damaged
cartilage in anteromedial gonarthrosis, a phenotype of
osteoarthritis. Osteoarthritis Cartilage.

[26] Saito T., Kawaguchi H. (2010). HIF-2α as a possible therapeutic target of
osteoarthritis. Osteoarthritis Cartilage.

[27] Su S., Dehnade F., Zafarullah M. (1996). Regulation of tissue inhibitor of metalloproteinases-3
gene expression by transforming growth factor-beta and
dexamethasone in bovine and human articular
chondrocytes. DNA Cell Biol.

[28] Pinho S., Niehrs C. (2007). Dkk3 is required for TGF-beta signaling during
*Xenopus* mesoderm
induction. Differentiation.

[29] Chou C.H., Lee C.H., Lu L.S., Song I.W., Chuang H.P., Kuo S.Y. (2013). Direct assessment of articular cartilage and
underlying subchondral bone reveals a progressive gene
expression change in human osteoarthritic knees. Osteoarthritis Cartilage.

[30] Zhu M., Tang D., Wu Q., Hao S., Chen M., Xie C. (2009). Activation of beta-catenin signaling in articular
chondrocytes leads to osteoarthritis-like phenotype in adult
beta-catenin conditional activation mice. J Bone Miner Res.

[31] Zhu M., Chen M., Zuscik M., Wu Q., Wang Y.J., Rosier R.N. (2008). Inhibition of beta-catenin signaling in articular
chondrocytes results in articular cartilage
destruction. Arthritis Rheum.

[32] Leijten J.C., Bos S.D., Landman E.B., Georgi N., Jahr H., Meulenbelt I. (2013). GREM1, FRZB and DKK1 mRNA levels correlate with
osteoarthritis and are regulated by osteoarthritis-associated
factors. Arthritis Res Ther.

[33] Dell'accio F., De Bari C., Eltawil N.M., Vanhummelen P., Pitzalis C. (2008). Identification of the molecular response of articular
cartilage to injury, by microarray screening: Wnt-16 expression
and signaling after injury and in osteoarthritis. Arthritis Rheum.

[34] Alexander S., Watt F., Sawaji Y., Hermansson M., Saklatvala J. (2007). Activin A is an anticatabolic autocrine cytokine in
articular cartilage whose production is controlled by fibroblast
growth factor 2 and NF-kappaB. Arthritis Rheum.

[35] van der Kraan P.M. (2014). Age-related alterations in TGF beta signaling as a
causal factor of cartilage degeneration in
osteoarthritis. Biomed Mater Eng.

[36] Hsu R.J., Lin C.C., Su Y.F., Tsai H.J. (2011). Dickkopf-3-related gene regulates the expression of
zebrafish myf5 gene through phosphorylated p38a-dependent Smad4
activity. J Biol Chem.

[37] Gruber J., Vincent T.L., Hermansson M., Bolton M., Wait R., Saklatvala J. (2004). Induction of interleukin-1 in articular cartilage by
explantation and cutting. Arthritis Rheum.

[38] Shen J., Li S., Chen D. (2014). TGF-beta signaling and the development of
osteoarthritis. Bone Res.

[39] van der Kraan P.M., van den Berg W.B. (2012). Chondrocyte hypertrophy and osteoarthritis: role in
initiation and progression of cartilage
degeneration?. Osteoarthritis Cartilage.

[40] Barrantes Idel B., Montero-Pedrazuela A., Guadano-Ferraz A., Obregon M.J., Martinez de Mena R., Gailus-Durner V. (2006). Generation and characterization of dickkopf3 mutant
mice. Mol Cell Biol.

[41] Kumon H., Sasaki K., Ariyoshi Y., Sadahira T., Ebara S., Hiraki T. (2015). Ad-REIC gene therapy: promising results in a patient
with metastatic CRPC following chemotherapy. Clin Med Insights Oncol.

